# DEG-BRIN-GCN: interpretable graph convolutional framework with differentially expressed genes brain region interaction network prior for AD diagnosis

**DOI:** 10.3389/fnins.2025.1697528

**Published:** 2025-12-11

**Authors:** Zhihao Zhang, Hui Liu, Lianghui Xu, Mo Sha, Ayiguli Halike, Wenzhong Yang, Ke Lv, Jingjing Wei

**Affiliations:** 1 School of Computer Science and Technology, Xinjiang University, Urumqi, China; 2 Institute of Medical Engineering Interdisciplinary Research, Xinjiang Medical University, Urumqi, China; 3 School of Public Health, Xinjiang Medical University, Urumqi, China

**Keywords:** Alzheimer's disease diagnosis, bioinformatics, graph convolutional networks, differential gene expression analysis, machine learning

## Abstract

Due to the intricate dynamic coupling between molecular networks and brain regions, early diagnosis and pathological mechanism analysis of Alzheimer's disease (AD) remain highly challenging. To address this, we propose a graph convolutional neural network framework (DEG-BRIN-GCN) based on a differentially expressed gene-brain region interaction network (DEG-BRIN), aiming to enhance both diagnostic accuracy and biological interpretability in AD research. We began by systematically analyzing transcriptomic data from 19 brain regions, identifying 329 differentially expressed genes that display widespread co-expression across multiple regions. Using these findings, we constructed DEG-BRIN to model prior associations among genes, thereby revealing potential molecular connectivity patterns implicated in AD pathological progression. Leveraging this network prior, we developed an AD classification model based on graph convolutional networks. Comparative experiments demonstrate that our proposed DEG-BRIN-GCN achieves significantly better diagnostic performance than three categories of baseline models: traditional machine learning methods, Random-GCN (models based on random network topologies), and PPI-GCN. Further analysis identified key brain regions–such as the superior parietal lobule, putamen, and frontal pole–along with high-contribution genes, including VCAM1, MCTP1, HBB, and CX3CR1, which play critical roles in AD pathology. Notably, this study is the first to implement a interpretability analysis based on a “gene-region-pathway” triad, offering a novel framework for cross-scale exploration of AD pathological mechanisms. Our findings underscore the central importance of inter-regional molecular interaction networks in the accurate diagnosis of AD.

## 1 Introduction

Alzheimer's Disease (AD) is a neurodegenerative disorder pathologically characterized by β-amyloid (Aβ) deposition and tau protein neurofibrillary tangles. Currently affecting over 55 million people globally, the AD patient population is projected to reach 152 million by 2050 (Alzheimer's Disease International, 2023). While cerebrospinal fluid (CSF) biomarkers and PET imaging support AD diagnosis (Zhu et al., 2023), their invasive nature and high costs limit clinical utility. Recent advances in plasma biomarkers have provided new opportunities for early screening (Lohman et al., 2024; Chiesa et al., 2020), yet their insufficient spatial resolution impedes the elucidation of brain region-specific molecular-network coupling mechanisms.

The brain-regional heterogeneity of gene regulatory networks provides novel insights into AD pathogenesis: Large-scale transcriptomic studies have revealed region-specific expression patterns of risk genes within default mode network regions (De Jager et al., 2018), while the propagation of pathological proteins along structural connectomes drives topological collapse of functional networks (Yu et al., 2021). These findings suggest potential couplings between molecular pathology and brain network dynamics in AD. However, current research has not adequately integrated cross-scale data, limiting mechanistic understanding and diagnostic precision.

In AD biomarker discovery and diagnostic modeling, differential gene analysis and weighted gene co-expression network analysis (WGCNA) have become pivotal approaches (Langfelder and Horvath, 2008; Madar et al., 2021; Zhang and Kiryu, 2023). Using key gene signatures as input features, machine learning models for AD diagnosis pioneered by Liu et al. (2023) have gained prominence. Jin et al. (2023) developed an enhanced deep learning algorithm with differentiable gene selection TabNet, demonstrating superior AD classification performance over traditional methods. Kalkan et al. (2022) mapped 1D gene expressions to 2D image-like representations via linear discriminant analysis for CNN-based classification. Hernández-Lorenzo et al. (2022) integrated gene interaction network topology with expression data for graph neural network-based phenotype prediction, validating biological prior integration. Nevertheless, most studies (Li et al., 2025; Zhou et al., 2025) rely on whole-brain averages or single-region expressions, neglecting inter-regional gene interaction patterns, thereby constraining model interpretability and generalizability.

In multimodal connectomics, imaging fusion techniques have significantly improved AD subtyping accuracy. The functionally-constrained structural graph variational autoencoder by Amodeo et al. (2022) enables cross-modal embedding of DTI and fMRI data. Wang et al. (2023) achieved AUC = 0.992 in subjective cognitive decline classification using metabolic-functional coupling indices. Furthermore, Lyu et al. (2024) constructed ultra-high-resolution connectomes based on tertiary hinged gyri, showing 12% AUC improvement in MCI classification over conventional parcellations, revealing micro-scale connection heterogeneity's diagnostic value. Despite these advances, current technologies still face molecular-network decoupling challenges, failing to address the fundamental question: “How do gene interactions drive network deterioration?”

This study addresses two core challenges in current AD research and clinical modeling. The first challenge is that existing AD diagnostic models often rely on whole-brain average gene expression or single-region expression data, which overlook the inter-regional co-occurrence patterns of differentially expressed genes (DEGs). The second challenge is that most models lack biological interpretability, as they fail to link molecular signatures (genes), spatial locations (brain regions), and pathological pathways to clarify the coupling mechanisms between molecular network abnormalities and brain region dysfunction in AD. To address these issues, this study proposes a graph convolutional network (GCN) framework, DEG-BRIN-GCN, built on the Differentially Expressed Gene-Brain Region Interaction Network (DEG-BRIN). The proposed framework not only integrates inter-regional gene interactions to enhance AD diagnostic performance but also improves biological interpretability through a “brain region-gene-pathway” triadic analysis framework, providing deeper insights into the molecular mechanisms of AD. The contributions of this study are as follows:

1. Differential gene mining and enrichment analysis: through mining and enrichment analysis of differential genes across 19 brain regions, this study reveals the brain region-specific expression patterns and functional pathways of AD-related genes. This provides molecular-level evidence for the pathophysiological mechanisms of AD.
2. Cross-brain region differential gene interaction network and graph neural network modeling: we constructed the DEG-BRIN based on the co-occurrence relationships of DEGs across brain regions. Using this network as prior knowledge, We developed a GCN-based diagnostic model for AD, significantly improving classification performance and offering a new technological approach for early AD diagnosis.
3. Triadic brain region-gene-pathway interpretability analysis: through triadic interpretability analysis integrating brain regions, genes, and pathways, we identified key brain regions and genes. Specifically:
  - CX3CR1 in the *putamen* drives motor dysfunction by regulating neurotransmitter transport and synaptic pruning.
  - High expression of VCAM1 in the *superior parietal lobule* is strongly associated with neuroinflammation and synaptic dysfunction.

**Figure 1:**
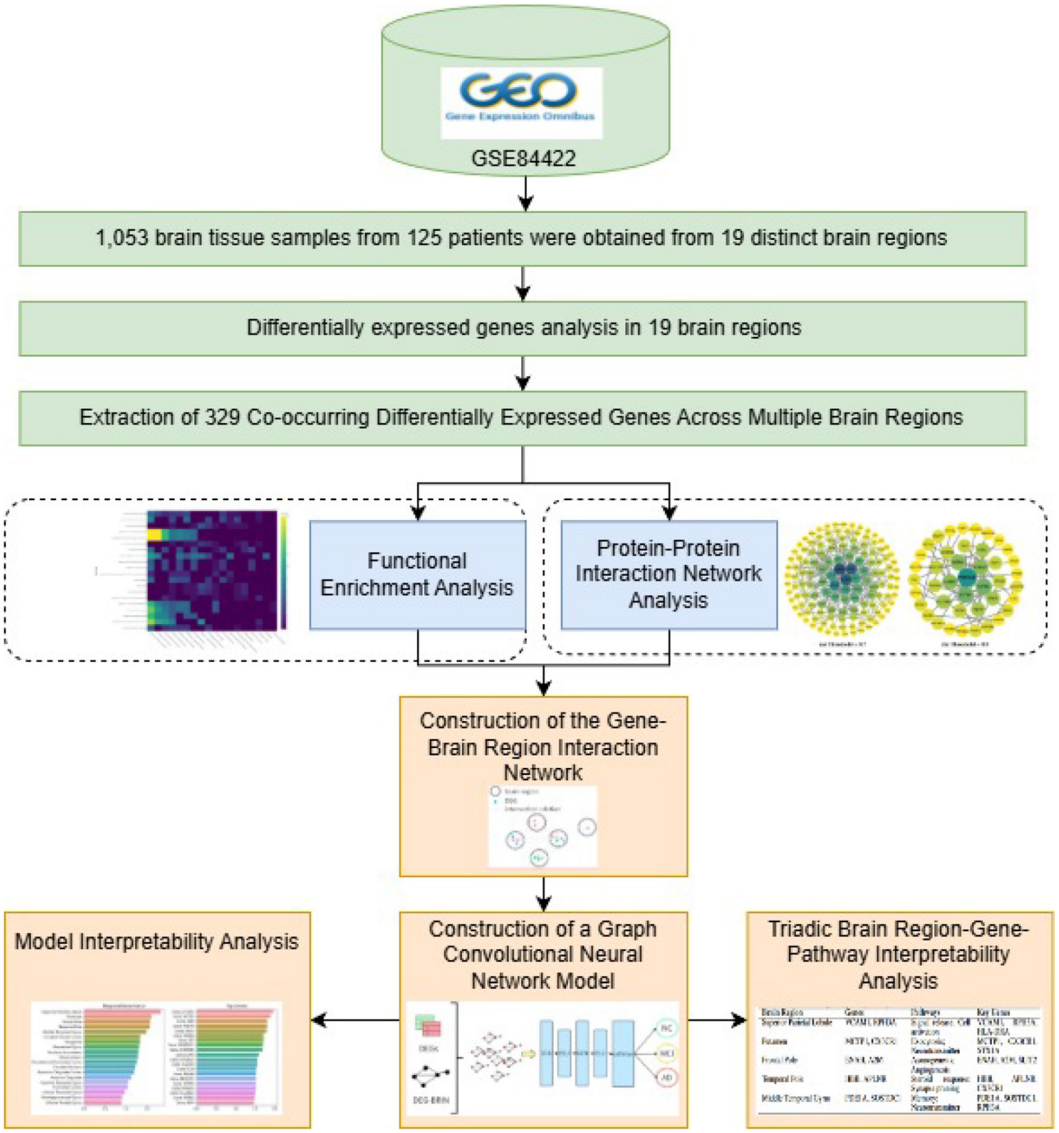
Paper framework diagram of AD diagnostic research.

These findings provide new insights into AD pathological mechanisms and biomarker discovery. The overall framework diagram of this paper is shown in Figure 1.

## 2 Data and methods

### 2.1 Data description

The dataset employed in this study, GSE84422, was retrieved from the NCBI Gene Expression Omnibus (GEO) database. It comprises 1,053 postmortem brain tissue samples collected from 125 individuals, with each subject contributing samples across 19 distinct anatomical brain regions. These regions were strategically selected to align with the well-documented “regional propagation” pattern of Alzheimer's disease (AD) pathology—which initially affects the medial temporal lobe and subsequently spreads to parietal, frontal, and other regions (Seeley et al., 2009)—thus covering critical stages of AD pathological progression. Specifically, the hippocampus and entorhinal cortex serve as early pathological targets, the superior parietal lobule and frontal pole represent intermediate-stage propagation regions, and the putamen and nucleus accumbens are late-stage affected regions. Such a selection ensures that differences in DEGs across these regions can reflect the “spatiotemporal heterogeneity of molecular network perturbations” during pathological progression, which is consistent with the temporal evolution characteristics of AD pathology. Samples were classified into four groups based on diagnostic criteria: Normal (*n* = 242), Possible AD (*n* = 193), Probable AD (*n* = 256), and Definite AD (*n* = 362).

### 2.2 Data preprocessing

The raw data from the GSE84422 dataset was acquired from the GEO database and preprocessed through a standardized pipeline. Initial data retrieval utilized the GEOquery package to download and partition samples across 19 brain regions. Probe-to-gene annotation was performed using the hgu133plus2.db database, during which probes lacking explicit gene annotations were filtered out. Genes containing missing values were systematically removed, and for genes mapped by multiple probes, the mean expression value was calculated to prevent redundant quantification. Stringent quality control removed genes with expression levels below detection thresholds in over 60% of samples, effectively reducing technical noise. The final preprocessed dataset comprised a standardized expression matrix of 18,925 protein-coding genes across 1,053 samples.

To investigate molecular signatures during the progression of AD, original diagnostic labels were consolidated into three analytical groups: Normal (CON, *n* = 242), Mild Cognitive Impairment (MCI, combining Possible AD and Probable AD, *n* = 449), and Definite AD (AD, *n* = 362). This stratification enabled three critical comparative analyses: (1) MCI vs. CON (MCI-CON) to identify early pathological molecular shifts, (2) AD vs. CON (AD-CON) to detect terminal-stage biomarkers, and (3) MCI vs. AD (MCI-AD) to pinpoint disease progression drivers. Each comparison targets distinct temporal phases of AD pathogenesis, from incipient network perturbations to end-stage neuropathological cascades.

### 2.3 Differential expression analysis

Transcriptomic profiles were analyzed using the *limma* package (v3.56.0) (Ritchie et al., 2015). To model the normalized expression values for each brain region *r*, we employed linear regression, formulated as:


$$\begin{array}{l} {\text{y}}_{r}={\text{X}}_{r}\beta_{r}+\epsilon_{r} \end{array}$$ (1)

Here, y_*r*_ represents the vector of normalized gene expression values across samples in brain region *r*. The design matrix X_*r*_ incorporates information such as diagnostic group assignments. The vector **β**_*r*_ contains the regression coefficients that quantify the influence of each component in X_*r*_ on gene expression. The residual term **ϵ**_*r*_ is assumed to follow a normal distribution $N\left(0,\sigma^{2}\text{I}\right)$, where I is the identity matrix, implying independent residuals with mean zero and constant variance σ^2^.

For the differential expression analysis, we utilized Empirical Bayes moderation to compute adjusted *t*-statistics, given by:


$$\begin{array}{l} {\tilde{t}}_{g}=\frac{{\hat{\beta }}_{g}}{{\tilde{s}}_{g}\sqrt{v_{g}}} \end{array}$$ (2)

In this statistic, ${\hat{\beta }}_{g}$ denotes the estimated regression coefficient for gene *g*, capturing the effect of a factor on the gene's expression. ${\tilde{s}}_{g}$ is the standard error of this coefficient, reflecting the uncertainty in its estimation, while *v*_*g*_ is a moderation factor derived via Empirical Bayes methods. This factor adjusts the standard error to enhance the robustness of the test, particularly in scenarios with small sample sizes.

In this study, DEGs were defined using a combination of criteria: a threshold of |log_2_FC| > 0.585 (corresponding to a 1.5-fold change in expression) along with a Benjamini-Hochberg adjusted *p*-value less than 0.05, which helps control the false discovery rate.

### 2.4 Functional enrichment analysis

Gene Ontology (GO) analysis was performed using *clusterProfiler* (Xu et al., 2024). The enrichment probability followed a hypergeometric distribution:


$$\begin{array}{l} P(X=k)=\frac{\left(\begin{array}{c} K\\ k \end{array}\right)\left(\begin{array}{c} N-K\\ n-k \end{array}\right)}{\left(\begin{array}{c} N\\ n \end{array}\right)} \end{array}$$ (3)

where *N* = background genes, *K* = genes in term *t*, *n* = DEGs.

### 2.5 Protein–protein interaction network analysis

The protein–protein interaction (PPI) network was constructed through a tiered confidence approach using the STRING database (Szklarczyk et al., 2023). Input genes for PPI network construction were the 329 cross-regional co-occurring DEGs (DEGs) identified in Section 2.3, ensuring the PPI network focused on molecular interactions relevant to cross-regional AD pathology. We first established the base network with medium-confidence interactions (*s* ≥ 0.4), corresponding to 50% probability of physical validation. Subsequent hierarchical filtering identified high-confidence hubs using thresholds of *s* ≥ 0.7 (70% confidence) and *s* ≥ 0.9 (90% confidence). The adjacency matrices were formally defined as:


$$\begin{array}{l} A_{ij}^{\left(k\right)}=\{\begin{array}{cc} 1 & \text{if }s_{ij}\geq \tau_{k}\\ 0 & \text{otherwise} \end{array} \end{array}$$ (4)

where i and j denote co-occurring genes, τ_*k*_ ∈ {0.4, 0.7, 0.9} represents tiered confidence thresholds and *s*_*ij*_ ∈ [0, 1] the combined interaction score. Topological analysis in Cytoscape (Otasek et al., 2019) computed centrality metrics across confidence tiers:


$$\begin{array}{ll} \text{Degree centrality:} & C_{D}\left(v\right)=\underset{u\in V}{\sum }A_{uv}^{\left(\tau \right)} \end{array}$$ (5)

$$\begin{array}{ll} \text{Betweenness centrality:} & C_{B}\left(v\right)=\underset{\begin{array}{c} s\neq v\neq t\\ s,t\in V \end{array}}{\sum }\frac{\sigma_{st}\left(v\right)}{\sigma_{st}} \end{array}$$ (6)

Here, *C*_*D*_(*v*) quantifies the number of direct protein interactors of node *v*, where *v* denotes a gene node in the PPI network. *C*_*B*_(*v*) represents the centrality of the difference, which is calculated using Equation 6. In Equation 6: σ_*st*_ denotes the total number of shortest paths from node *s* (a gene node in the PPI network) to node *t* (a gene node in the PPI network); σ_*st*_(*v*) denotes the number of shortest paths from node *s* to node *t* that pass through node *v* (the target gene node). Hub genes are defined as genes for which both *C*_*D*_(*v*) and *C*_*B*_(*v*) rank in the top 20% of all nodes at each confidence tier. Through this tiered filtering strategy, 132 hub genes were identified at τ = 0.7, and 42 high-confidence hub genes were retained at τ = 0.9.

### 2.6 DEG-BRIN interpretable graph convolutional framework

**Figure 2:**
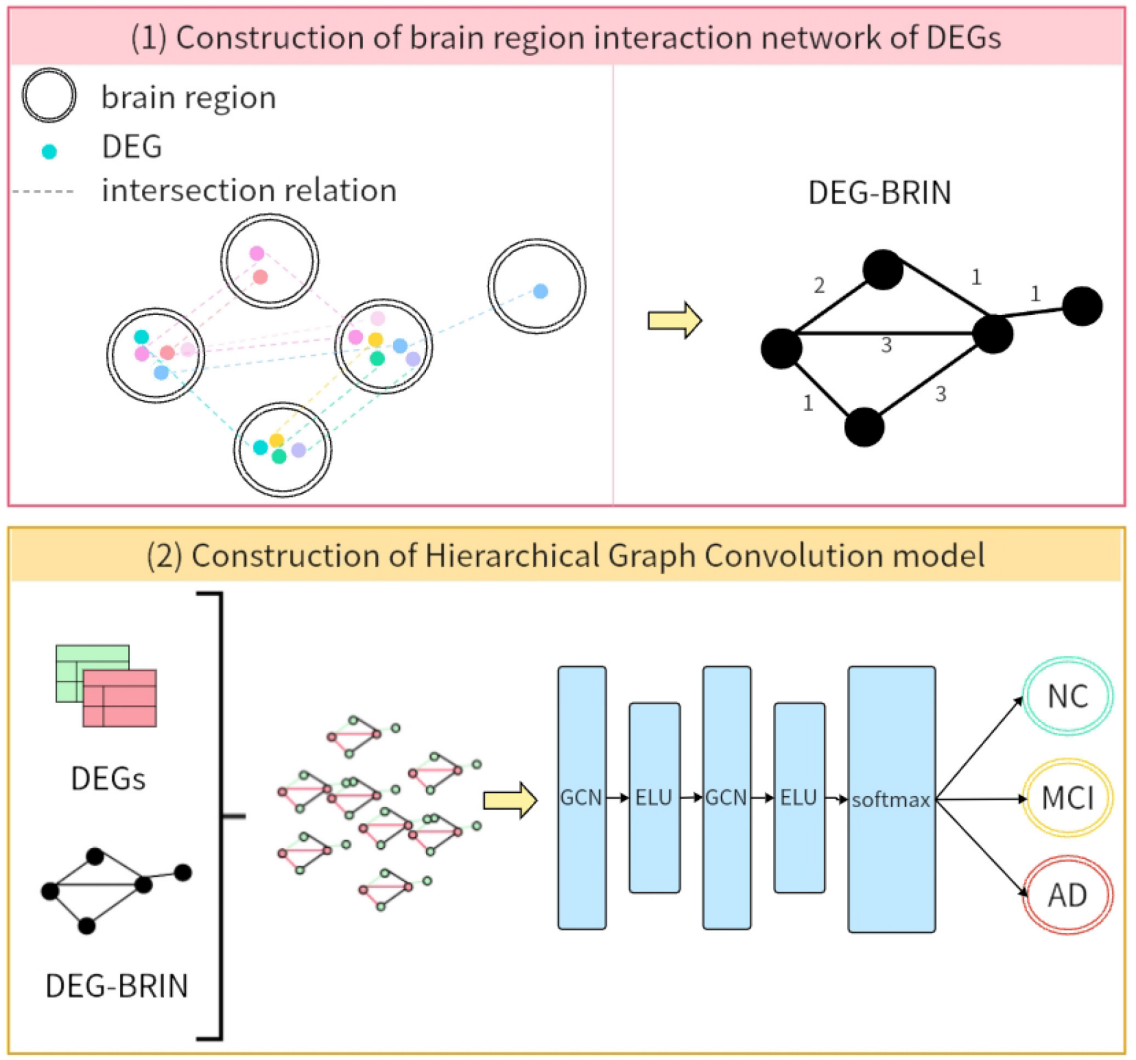
DEG-BRIN-GCN framework for AD diagnosis.

The DEG-BRIN-GCN framework integrates bioinformatics-derived molecular signatures by leveraging DEG-BRIN, which encodes cross-regional gene co-occurrence patterns, and combines it with biologically-constrained graph convolution to achieve interpretable AD diagnosis. The framework architecture, as illustrated in Figure 2, comprises two components (i.e., DEG-BRIN construction and hierarchical GCN modeling), with design logic tailored to address AD's pathological and data characteristics, as detailed below.

#### 2.6.1 Graph construction

Let G=(V,E,W) denote an undirected brain-region graph where W specifically serves as the weighted adjacency matrix of DEG-BRIN, $V=\left\{u_{1},\ldots ,u_{19}\right\}$ indexes 19 anatomical regions profiled in this study. For each region *u*_*i*_ we compile a region-specific DEG set $G_{i}=\left\{g_{k}\in G|\;g_{k}\text{is significantly dys-regulated in}u_{i}\;\right\}$, where the universal gene list $G=\left\{g_{k}|N_{k}\geq 2\right\}$ retains only genes that are differentially expressed in at least two regions [multifocal pathology hypothesis(Seeley et al., 2009)].

An edge $e_{ij}\in E$ is established iff *G*_*i*_ ∩ *G*_*j*_ ≠ ∅, and its weight quantifies the size of the shared signature:


$$w_{ij}=|G_{i}\cap G_{j}|.$$

Self-loops (*w*_*ii*_ = |*G*_*i*_|) are added to preserve regional self-information.

#### 2.6.2 Hierarchical graph convolution model

We use the average log_2_-CPM(Counts Per Million) for feature initialization, mainly because the dataset has undergone strict preprocessing–genes with expression levels below detection thresholds in >60% of samples, missing values, and redundant probes were systematically removed. This preprocessing minimized extreme outliers, allowing the average to better retain the overall expression levels of DEGs. Two graph convolution layers with exponential linear units (ELU) refine representations:


$$\begin{array}{l} {\text{H}}^{\left(1\right)}=\mathrm{ELU}(\hat{\text{A}}{\text{H}}^{\left(0\right)}\Theta^{\left(1\right)}), \end{array}$$ (7)

$$\begin{array}{l} {\text{H}}^{\left(2\right)}=\mathrm{ELU}(\hat{\text{A}}{\text{H}}^{\left(1\right)}\Theta^{\left(2\right)}), \end{array}$$ (8)

where **Θ**^(1)^ ∈ ℝ^329×256^ and **Θ**^(2)^ ∈ ℝ^256×256^ are trainable weight matrices. Layer normalization is applied before the final pooling to preserve anatomical interpretability.

A graph-level signature is obtained via global mean pooling: $\text{z}=\frac{1}{19}\overset{19}{\underset{i=1}{\sum }}{\text{h}}_{i}^{\left(2\right)}\in {\mathbb{R}}^{256}$, and mapped to diagnostic probabilities by a softmax classifier:


$$\hat{\text{y}}=\mathrm{softmax}({\text{W}}_{c}\text{z}+{\text{b}}_{c}),\;{\text{W}}_{c}\in {\mathbb{R}}^{3\times 256}.$$

The model is trained with cross-entropy loss and early stopping (patience = 20 epochs).

### 2.7 AD diagnostic model construction based on DEG-BRIN-GCN

The graph structural knowledge required for the model is derived from the co-occurrence relationships of DEGs across different brain regions in multiple control experiments. Ideally, the graph model should be constructed using gene expression data from 19 brain regions of a single individual. However, due to limited sample size, we apply the gene expression data from one brain region of an individual to the entire graph model for modeling.

To ensure reliable model evaluation and generalizability, a stratified sampling strategy combined with random over-sampling (ROS) was implemented to address class imbalance. The original dataset was partitioned into training (70%, *n* = 737), validation (10%, *n* = 105), and test sets (20%, *n* = 211), with ROS applied exclusively to the training set to preserve the natural data distribution in validation and testing phases.

This diagnostic model employs a two-layer GCN architecture with 329-dimensional input features, aiming to prevent the excessive smoothing of spatial correlation information between brain regions. An exponential linear unit (ELU) activation function is used between the graph convolution layers to maintain stable training of the model on brain region data. Overfitting is mitigated by adding a dropout layer (probability = 0.5). Critical biological interpretability was enhanced through trainable parameter matrices encoding regional importance (19-dimensional) and gene significance (329-dimensional).

For hyperparameter optimization, the Optuna framework with tree-structured Parzen estimator (TPE) sampling was utilized to efficiently explore the parameter space. The search domain encompassed hidden layer dimensions (64–256 units), learning rates (1 × 10^−4^ to 1 × 10^−3^), weight decay coefficients (1 × 10^−6^ to 1 × 10^−3^), and dropout probabilities (0.3–0.7). After 30 optimization trials, the optimal configuration achieved a validation accuracy of 0.9185 with hidden_size = 103, lr = 0.00703, dropout = 0.458, and weight_decay = 1.06 × 10^−5^.

## 3 Results and analysis

### 3.1 Differential gene expression analysis results

**Figure 3:**
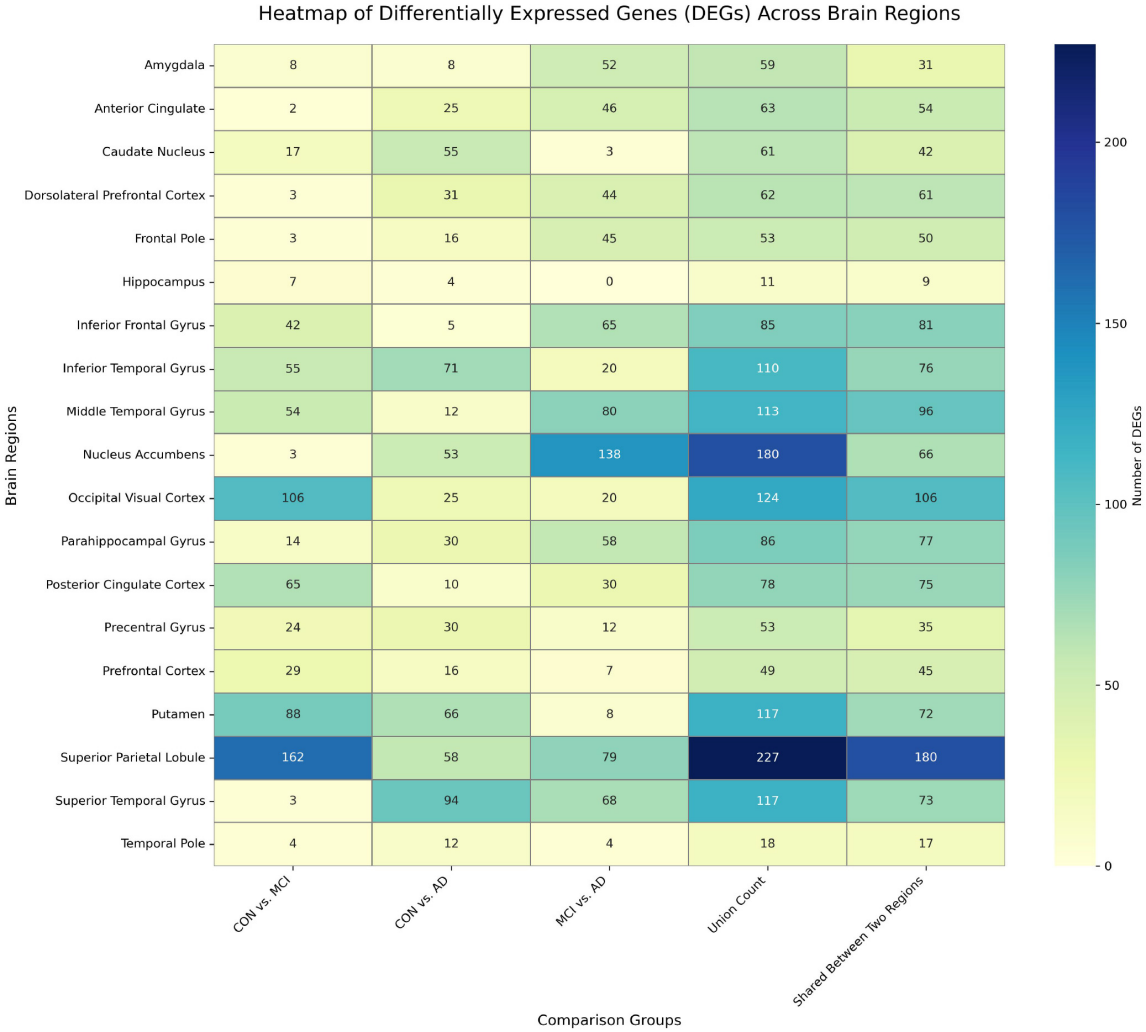
Heatmap of the number of DEGs across each brain region for different comparisons.

We performed DGE analysis across 19 brain regions for three pairwise experimental groups: MCI vs. CON, AD vs. CON, and MCI vs. AD. The distribution of DEGs is presented in Figure 3. To ensure the robustness of the findings, we computed the union of the results from these three group comparisons, yielding a consolidated set of DEGs across the 19 brain regions. Detailed data are reported in the “Union Count” column of Figure 3. The heatmap of DEG counts across different brain regions highlights significant variability, underscoring the regional heterogeneity of molecular changes associated with AD.

For the CON_MCI group, the superior parietal lobule exhibited the highest number of DEGs (162), followed by the occipital visual cortex (106), whereas the anterior cingulate showed only two DEGs. This distribution suggests region-specific transcriptional characteristics during the MCI stage. In the MCI_AD group, the nucleus accumbens displayed the highest DEG count (138), while notably, no DEGs were detected in the hippocampus (0). In the union analysis of DEGs, the *Superior Parietal Lobule* exhibited the highest number of DEGs (227), while the *hippocampus* showed a relatively low count (11). This distinct spatial distribution indicates the superior parietal lobule's potential critical molecular role in AD progression, whereas the hippocampal gene expression changes may stabilize during late-stage AD pathogenesis, potentially reflecting region-specific molecular dynamics in neurodegeneration.

**Figure 4:**
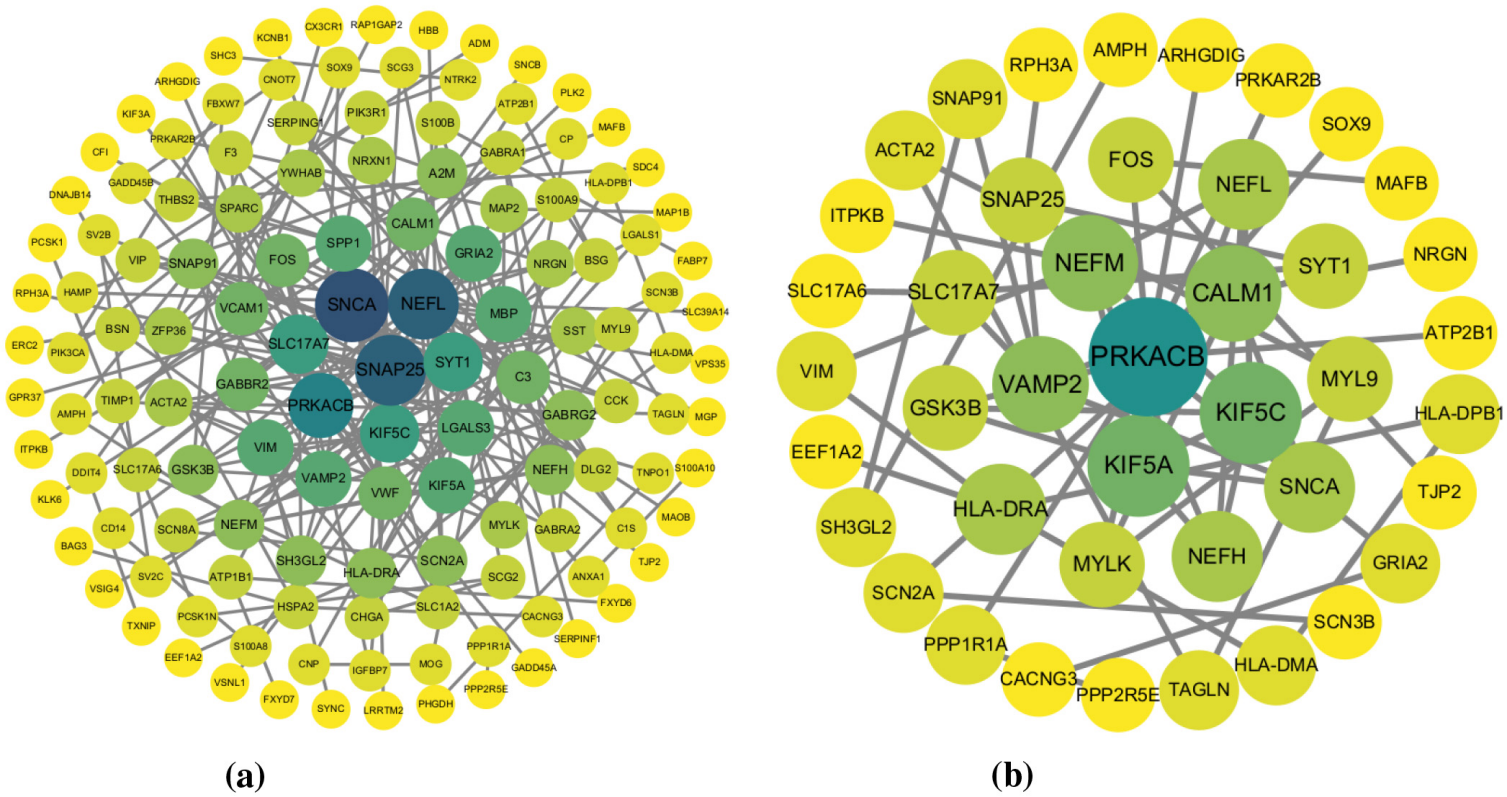
PPI Networks of shared DEGs with different thresholds: **(a)** 0.7, **(b)** 0.9.

We identified 329 shared DEGs that co-occurred in two or more brain regions (frequency ≥2), suggesting potential inter-regional molecular connectivity. Protein–protein interaction (PPI) network analysis of these shared DEGs revealed 132 hub genes at a confidence threshold of 0.7, with 42 high-confidence hub genes retained at the more stringent threshold of 0.9. These hub gene networks were visualized using Cytoscape (Figure 4). The 42 high-confidence hubs retained at STRING ≥ 0.9 are significantly enriched in microglial- and synaptic-function genes (e.g., CX3CR1, RPH3A, SYT1), implying that the most robust inter-regional molecular links involve neuroinflammatory and vesicle-trafficking pathways rather than broad transcriptional noise.

**Figure 5:**
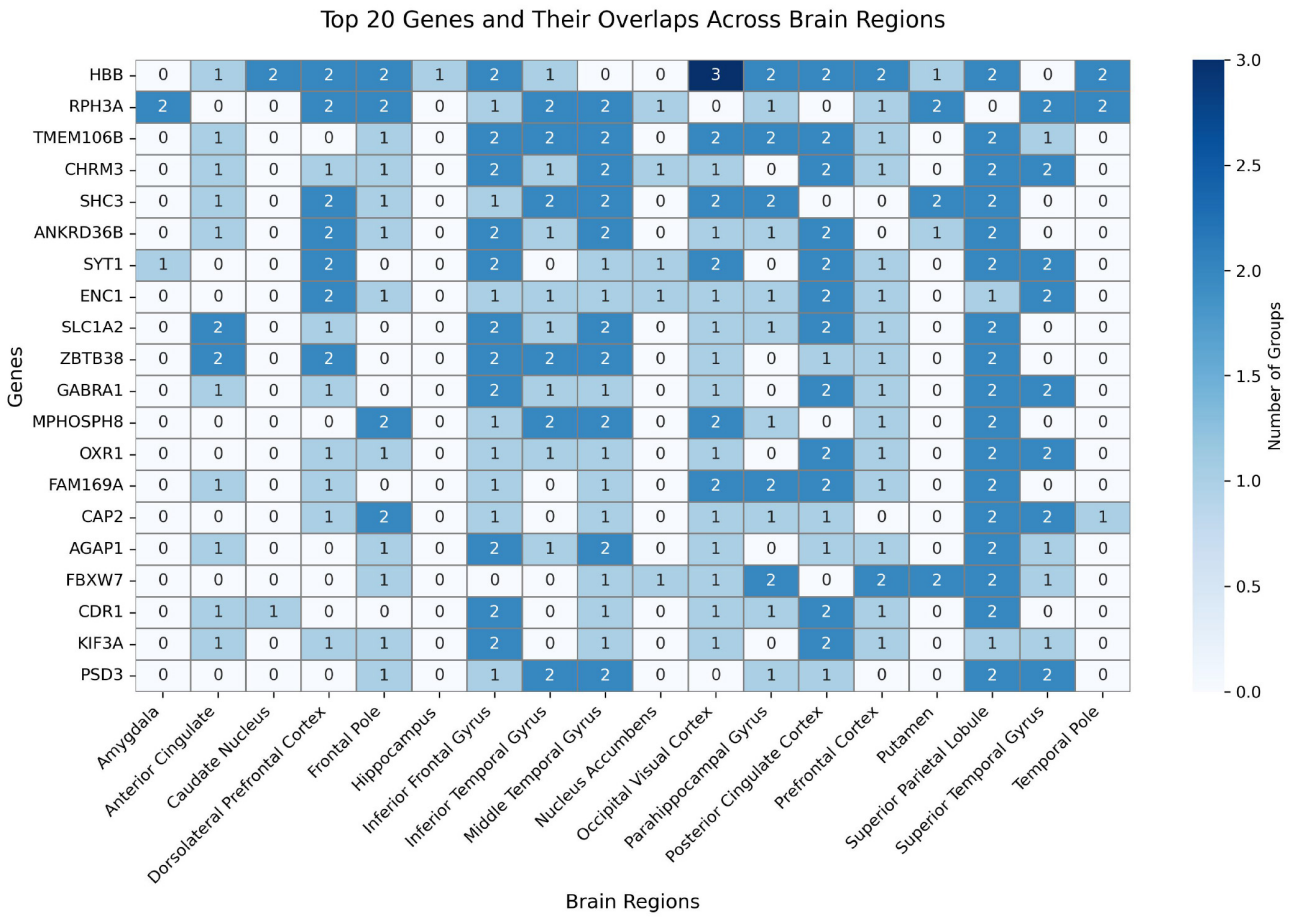
Heatmap of top 20 active genes across brain regions in three experimental comparisons.

Subsequent analysis focused on the top 20 most frequently occurring DEGs across all brain regions. A heatmap depicting their expression frequency patterns in the three experimental comparisons (MCI vs. CON, AD vs. CON, and MCI vs. AD) is presented in Figure 5, revealing distinct spatial-temporal activation profiles associated with disease progression. The high total frequency of HBB, RPH3A, and TMEM106B suggests that they are not confined to a single stage but are repeatedly activated in multiple differential pathways, indicating that their functions may be involved in cross-stage shared pathological mechanisms.

### 3.2 Pathway enrichment analysis of region-specific degs

**Figure 6:**
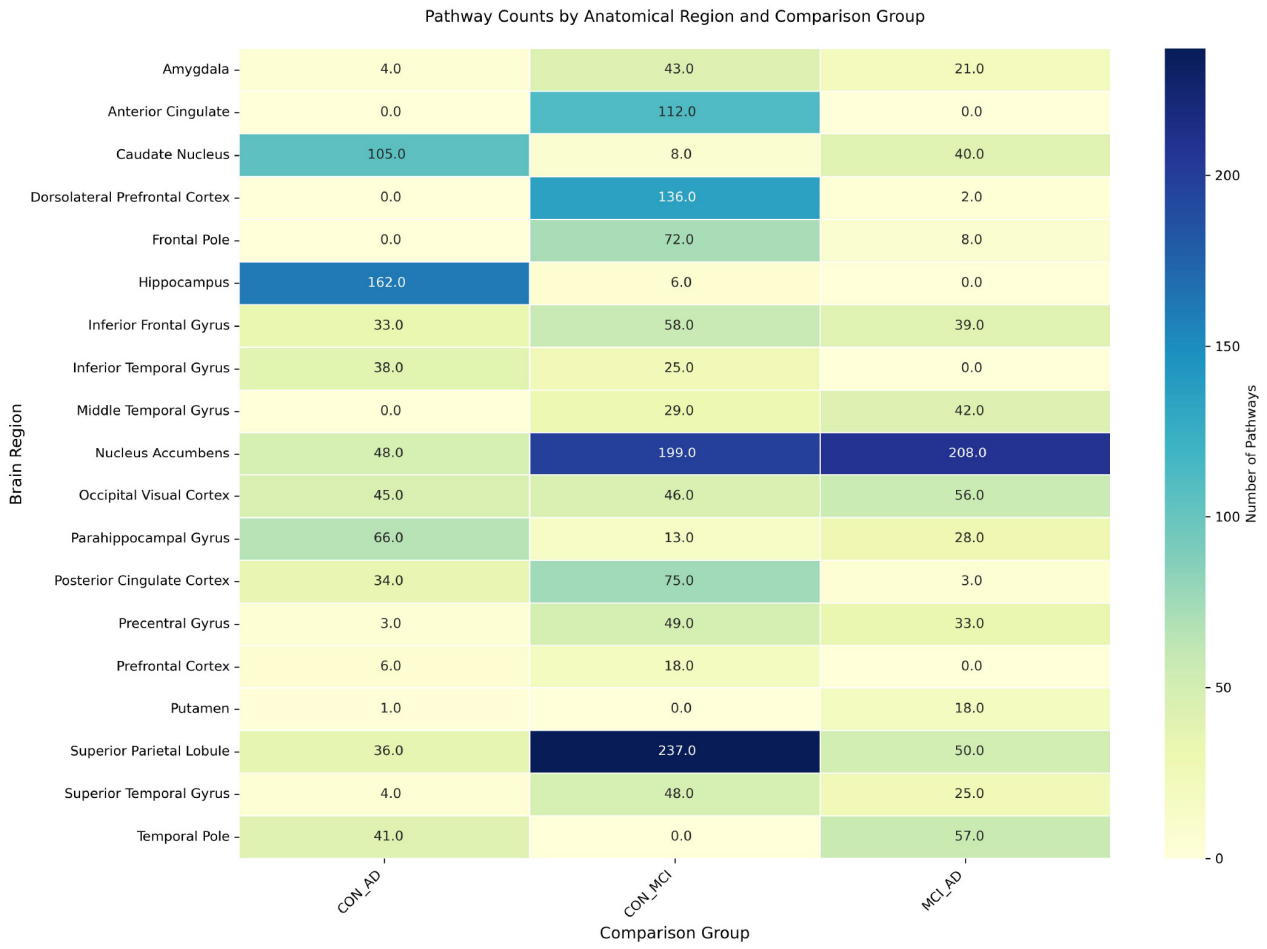
Heatmap of pathway enrichment counts for DEGs across brain regions.

**Figure 7:**
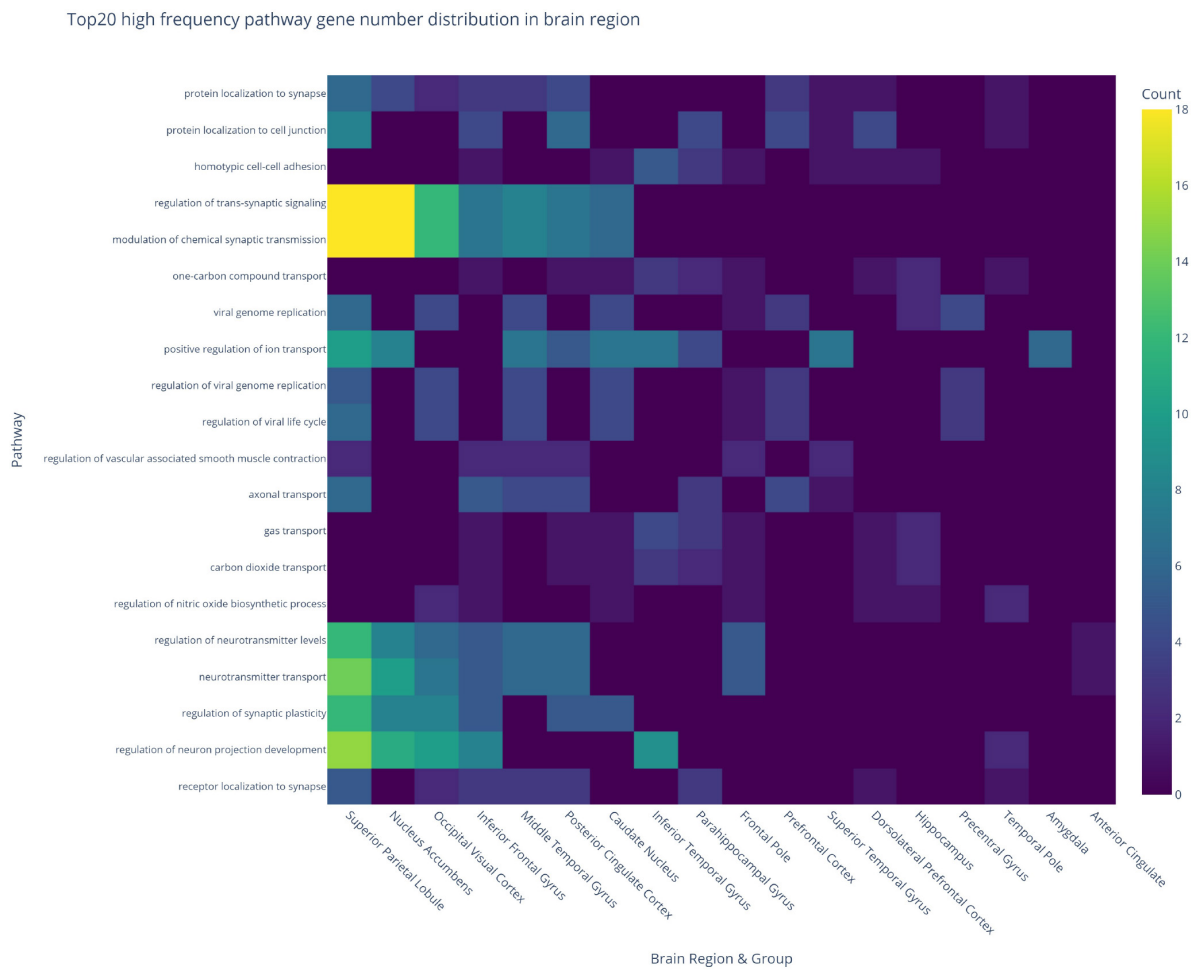
Heatmap of top 20 high-frequency pathway gene distribution across brain regions.

To investigate the molecular mechanisms underlying neurodegeneration, pathway enrichment analysis was performed on DEGs across 19 brain regions and three experimental comparisons (CON_MCI, MCI_AD, CON_AD), identifying 2,430 significantly enriched pathways. The heatmap in Figure 6 displays the regional distribution of enriched pathways across comparison groups. The superior parietal lobule exhibited the most prominent enrichment in the CON_MCI group (237 pathways), while the nucleus accumbens showed abnormally high pathway counts in both MCI_AD (208 pathways) and CON_MCI (199 pathways) comparisons. Notably, the dorsolateral prefrontal cortex demonstrated substantial pathway enrichment in the CON_MCI group (136 pathways). Despite the relatively low number of DEGs identified in the hippocampus, its associated genes displayed the highest pathway enrichment (162 pathways) in the CON_AD group, suggesting exceptional molecular complexity indicative of its role as a core pathological target in early-stage AD. Analysis of the top 20 most frequently enriched pathways across brain regions (Figure 7) revealed synaptic dysregulation as the predominant functional category. The superior parietal lobule (18 genes), nucleus accumbens (18 genes), and occipital visual cortex (12 genes) showed the highest gene counts for pathways regulating synaptic signaling and chemical synaptic transmission, with additional detections in the inferior frontal gyrus (seven genes) and middle temporal gyrus (eight genes). Neurotransmitter transport pathways peaked in the superior parietal lobule (14 genes) and nucleus accumbens (10 genes), with moderate enrichment observed in the occipital visual cortex (seven genes) and prefrontal cortex (five genes). Synaptic plasticity regulation pathways were prominent in the superior parietal lobule (12 genes), nucleus accumbens (eight genes), and occipital visual cortex (eight genes). Region-specific neuroinflammatory patterns emerged, with viral genome replication and viral life cycle regulation pathways most active in the superior parietal lobule (six genes each) and caudate nucleus (four genes each). Vascular dysfunction markers, particularly pathways regulating vascular smooth muscle contraction, were enriched in the inferior frontal gyrus (two genes) and middle temporal gyrus (two genes).

### 3.3 Comparative experimental results

A comprehensive evaluation of DEG-BRIN-GCN performance was conducted through systematic comparisons from multiple perspectives. Traditional machine learning models were first established as baseline comparators to assess DEG-BRIN-GCN's advantages in processing high-dimensional features and complex relational data. Subsequent ablation studies with alternative graph structures were designed to validate the effectiveness of brain region interaction graphs. The PPI-GCN control model, constructed using protein–protein interaction networks (nodes = genes, edges = PPI relationships), evaluated gene-level interaction contributions to AD classification. The Random-GCN model, featuring randomly generated nodes with equivalent gene counts and edge numbers to DEG-BRIN-GCN, served to verify information gain from brain network topology.

Table 1 presents the classification accuracy of different models on the independent test set, traditional machine learning models demonstrated varied test set performance. Logistic Regression and XGBoost achieved peak accuracy (0.76), while GaussianNB (0.521) and Decision Tree (0.441) underperformed. Random Forest showed intermediate capability (0.678 accuracy). The DEG-BRIN-GCN model has the best performance, with an accuracy rate of 0.839. The random-GCN model has a moderate accuracy rate (0.77), which is close to the level of traditional machine learning, confirming the value of information from the topological structure of the brain network. Notably, PPI-GCN model exhibits a certain level of diagnostic capability, yet with limited efficacy, achieving an accuracy of 0.82 on the test set–slightly lower than that of the DEG-BRIN-GCN proposed in this study. Specifically, PPI-GCN is solely based on protein–protein interactions and ignores spatial associations between brain regions. In contrast, DEG-BRIN integrates cross-regional gene co-occurrence patterns, which is more consistent with the “regional propagation” characteristic of AD pathology. Consequently, DEG-BRIN-GCN demonstrates superior performance.

**Table 1:** Performance comparison of different models on the test set.

**Model**	**AD**			**MCI**			**Normal**			**Acc**±**SD**
	**P**	**R**	**F1**	**P**	**R**	**F1**	**P**	**R**	**F1**	
GaussianNB	0.62	0.54	0.58	0.47	0.52	0.50	0.44	0.48	0.46	0.52 ± 0.01
XGBoost	0.77	0.83	0.80	0.81	0.74	0.77	0.83	0.79	0.81	0.79 ± 0.02
SVM	0.78	0.77	0.77	0.79	0.71	0.75	0.66	0.77	0.71	0.75 ± 0.02
Logistic	0.79	0.74	0.77	0.86	0.77	0.81	0.61	0.77	0.68	0.76 ± 0.01
Random-GCN	0.81	0.76	0.78	0.81	0.85	0.83	0.68	0.71	0.69	0.77 ± 0.04
PPI-GCN	0.83	0.68	0.75	0.77	0.89	0.82	0.86	0.84	0.85	0.82 ± 0.02
DEG-BRIN-GCN	0.86	0.89	0.87	0.87	0.82	0.85	0.78	0.79	0.78	0.84 ± 0.02

p (precision), real positives among predicted positives; R (recall), correctly predicted real positives; F1 (F1-score), harmonic mean of P and R; Acc (accuracy), correctly predicted total samples (overall classification ability); SD, standard deviation.

### 3.4 Interpretability analysis

**Figure 8:**
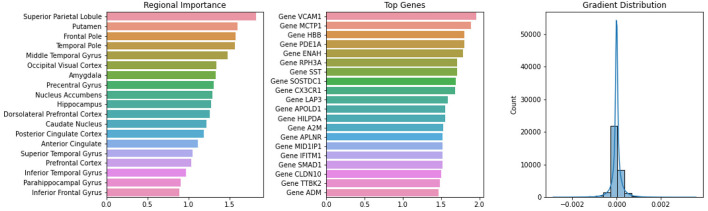
Regional transcriptomic signatures of AD. Heatmap visualization of DEGs across brain regions, with hierarchical clustering demonstrating region-specific expression patterns.

Integrated visualization modules for regional importance, gene significance, and gradient distributions were implemented during model training to elucidate biological interpretability. As shown in Figure 8, the heatmap analysis revealed distinct spatial patterns of across brain regions, providing a transcriptional landscape of AD pathology.

Regional importance analysis identified the superior parietal lobule, putamen, and frontal pole as the most contributive brain regions for AD classification through quantitative bar plot rankings. These regions, known for their involvement in amyloid-β aggregation, tau propagation, and synaptic loss in AD pathogenesis, demonstrate the model's ability to detect functionally relevant connectivity patterns associated with disease progression.

Gene significance analysis revealed a prioritized set of 20 AD-associated biomarkers through feature importance rankings. Remarkably, 50% of these top-ranked genes (*VCAM1, HBB, PDE1A, RPH3A, SST, CX3CR1, A2M, APLNR, IFITM1*, and *ADM*) were concurrently identified as hub nodes in the protein–protein interaction (PPI) network analysis at the τ = 0.7 confidence threshold (Equation 4). This convergence between machine-learned feature importance and biological network centrality underscores the mechanistic plausibility of our feature selection framework. The model highlighted several pathologically significant genes, including *VCAM1* (neuroinflammation and vascular dysfunction), *MCTP1* (calcium homeostasis), and *HBB* (hypoxia response), which align with established AD pathways. Further validation emerged through the identification of *PDE1A* (cAMP-mediated signaling), *ENAH* (cytoskeletal dynamics), *RPH3A* (synaptic plasticity), and *SST* (neuroendocrine regulation), all exhibiting strong AD literature support. Additional candidates such as *CX3CR1* (microglial activation), *APOLD1* (vascular remodeling), *HILPDA* (lipid metabolism), and *A2M* (proteostasis) demonstrated coherent pathway associations. Novel mechanistic insights were provided by the identification of *SMAD1* (TGF-β signaling cascades), *CLDN10* (blood-brain barrier integrity), and *TTBK2* (neuronal microtubule dynamics), suggesting promising targets for therapeutic development.

The training stability was quantitatively verified through gradient distribution analysis, revealing tightly bounded parameter updates ranging from −0.0029 to 0.0036 (mean = 0.0000 ± 0.0003). The symmetric, leptokurtic distribution (kurtosis = 3.1) centered at zero confirmed effective mitigation of gradient vanishing/explosion phenomena. This stable optimization landscape underscores the model's capacity to learn complex neuropathological relationships while maintaining numerical robustness throughout the training process.

### 3.5 Triadic brain region-gene-pathway interpretability analysis

Through integrative analysis of brain network topology, key gene signatures, and pathway enrichment results, this study elucidates cross-scale mechanisms linking brain regions, genes, and pathways in AD progression, as detailed in Table 2.

**Table 2:** Enriched brain regions, genes, pathways, and key genes.

**Brain region**	**Genes**	**Pathways**	**Key genes**
Superior parietal lobule	VCAM1, RPH3A	Signal release; cell activation	VCAM1, RPH3A, HLA-DRA
Putamen	MCTP1, CX3CR1	Exocytosis; neurotransmitter	MCTP1, CX3CR1, STX1A
Frontal pole	ENAH, A2M	Axonogenesis; angiogenesis	ENAH, A2M, SLIT2
Temporal pole	HBB, APLNR	Steroid response; synapse pruning	HBB, APLNR, CX3CR1
Middle temporal gyrus	PDE1A, SOSTDC1	Memory; neurotransmitter	PDE1A, SOSTDC1, RPH3A

The superior parietal lobule exhibited coordinated pathological contributions through *VCAM1*-mediated positive regulation of cell activation (via *HLA-DRA* and *TYROBP*) alongside *RPH3A*-driven synaptic signaling pathways (*STX1A, SYT1*). In the putamen, *MCTP1* and *CX3CR1* were respectively associated with exocytosis mechanisms (*STX1A, VAMP8*) and neurotransmitter transport pathways (*SLC17A7, SLC38A1*). The frontal pole demonstrated dual pathological axes featuring *ENAH*-mediated axonogenesis (*SLIT2, MAP1B*) and *A2M*-regulated vascular angiogenesis (*APLNR, C3*).

Notably, *CX3CR1* emerged as a multi-regional regulator, showing significant enrichment across four brain regions including the superior parietal lobule and temporal pole. This pleiotropic gene orchestrated synaptic pruning through *C3* and *ITPKB*, while concurrently modulating angiogenesis via *APLNR* and *AQP1*. The spatial convergence of *CX3CR1*-associated pathways underscores its pivotal role in bridging neuroinflammatory and vascular components of AD pathology.

## 4 Discussion

This study introduces a DEG-BRIN framework for graph convolutional networks, derived from differential gene co-occurrence patterns across brain regions. The proposed DEG-BRIN-GCN model achieved superior classification accuracy (0.839) compared to traditional machine learning approaches (peak accuracy = 0.76 for XGBoost/Logistic Regression). PPI-GCN achieved a slightly lower accuracy of 0.82. While effective for functional genomics research, this model fails to adequately capture the spatial associations between brain regions, which limits its performance in AD diagnosis that relies on integrating cross-regional molecular patterns. Though effective for functional genomics research, it still inadequately captures the spatial associations between brain regions. The integration of neurobiological priors through DEG-BRIN demonstrates significant potential for multi-scale biological network modeling, overcoming limitations of conventional graph neural networks reliant on unimodal topological data.

The three-dimensional brain region-gene-pathway analytical system, constructed from DEG-BRIN-GCN's importance outputs, revealed mechanistically coherent associations. CX3CR1 in the putamen was found to regulate neurotransmitter transport (*SLC17A7, SLC38A1*) and synaptic pruning (C3 pathway), corroborating experimental evidence from Pei et al. (2024) showing CX3CR1 knockout-induced motor deficits (30% reduction in rotarod performance) and 5-HTR2a downregulation (*p* < 0.001). These findings position the putaminal CX3CR1-microglial axis as a potential core mechanism for AD-related motor symptoms. Furthermore, VCAM1 overexpression in the superior parietal lobule showed dual associations with neuroinflammation and synaptic dysfunction, aligning with cognitive improvements observed following VCAM1 suppression in AD mouse models (Zhu et al., 2025).

Despite these advances, several limitations require further consideration. First, the foundational DEG-BRIN framework of this study is entirely derived from postmortem brain tissue data of 125 subjects. A key limitation of this data source is the limited number of multi-region samples per individual–which not only makes it difficult to distinguish between intra-subject regional synergy and inter-subject variation but also may increase the risk of overfitting. Second, the model cannot undergo rigorous external validation: on the one hand, we only used the GSE84422 dataset, which limits the validation of the model's generalizability; on the other hand, the “postmortem” nature of this dataset fails to capture the dynamic pathological processes of AD in living organisms. Third, the stratification of sample analysis groups does not cover the very early stage of AD: the MCI group in this study was formed by merging “Possible AD” and “Probable AD” samples, while Subjective Cognitive Decline (SCD) samples–widely recognized as the “pre-MCI” or very early stage of AD—were not included in the analysis. This omission not only leaves the diagnostic performance of the DEG-BRIN-GCN model for SCD unevaluated but also fails to address the critical question of whether the model can detect subtle cross-regional molecular perturbations before the onset of cognitive impairment—a capability essential for the early intervention of AD.

To address these limitations, future improvements to the framework should prioritize the following directions: Methodologically, WGCNA can be integrated to replace the current differential gene co-occurrence weighting paradigm, thereby identifying “conserved cross-regional modules.” Such modules can more accurately reflect the robust molecular networks underlying AD. Meanwhile, the implementation of L1 graph regularization can retain only high-frequency and strong connections in DEG-BRIN, reducing noise from weak or spurious associations, and thus improving the model's biological plausibility and generalization ability. Data-wise, expanding to multi-source datasets is crucial—future work should seek or generate more multi-brain-region AD datasets to validate the model's performance across different populations. Additionally, combining postmortem transcriptomic data with *in vivo* data enables the model to capture the dynamic pathological processes of AD and bridge the gap between molecular mechanisms and clinical phenotypes in living patients. Clinically, including SCD samples in future analyses is essential for evaluating the model's ability to diagnose very early-stage AD: by classifying SCD, Possible AD, and Probable AD into distinct subgroups, we can verify whether the DEG-BRIN-GCN can detect stage-specific cross-regional molecular signatures, ultimately enhancing its utility in early AD intervention and disease monitoring.

## 5 Conclusion

This study introduces DEG-BRIN-GCN, an interpretable graph convolutional framework that integrates gene expression data with brain network topology to achieve precise disease classification, while providing mechanistic insights through a novel gene-region-pathway triad interpretability paradigm. Benchmarking experiments demonstrate that the framework functions not only as a computationally robust diagnostic tool but also as a biologically plausible model for understanding the pathogenesis of Alzheimer's disease. The analysis identifies VCAM1-mediated neuroinflammatory pathways in the superior parietal lobule and CX3CR1-regulated synaptic pruning in the putamen as key pathophysiological hubs in Alzheimer's disease. The three-scale interpretability—spanning molecular signatures, regional vulnerability patterns, and system-level pathway disruptions—offers a transformative perspective for neurodegenerative disease research and paves the way for the development of precision therapeutics.

## Data Availability

The original contributions presented in the study are included in the article/supplementary material, further inquiries can be directed to the corresponding authors.
